# Strategies for screening cord blood for a public cord blood bank in high HIV prevalence regions

**DOI:** 10.1017/gheg.2018.6

**Published:** 2018-05-15

**Authors:** M. Meissner-Roloff, L. Gaggia, M. Vermeulen, A. F. H. Mazanderani, N. M. du Plessis, H. C. Steel, M. S. Pepper

**Affiliations:** 1 Department of Immunology, Institute for Cellular and Molecular Medicine, Faculty of Health Sciences, and SAMRC Extramural Unit for Stem Cell Research and Therapy, University of Pretoria, Pretoria, South Africa; 2 South African National Blood Service, 1 Constantia Boulevard, Constantia Kloof Ext. 22, Weltevreden Park, South Africa; 3 National Institute for Communicable Diseases, National Health Laboratory Services, 1 Modderfontein Road, Sandringham, South Africa; 4 Department of Medical Virology, Faculty of Health Sciences, University of Pretoria, Pretoria, South Africa; 5 Department of Paediatrics, Kalafong Provincial Tertiary Hospital and Faculty of Health Sciences, University of Pretoria, Private Bag X396, Pretoria, South Africa

**Keywords:** Africa, cord blood, HIV, stem cell, transplantation, ART, anti-retroviral therapy, CMV, cytomegalovirus, DNA, deoxyribonucleic acid, ELISA, enzyme-linked immunosorbent assay, EPI, Expanded Programme of Immunisation, FDA, Food and Drug Administration, HBV, hepatitis B virus, HCV, hepatitis C virus, HIV, human immunodeficiency virus, HLA, human leukocyte antigen, HSCT, haematopoietic stem cell transplantation, MTCT, mother-to-child transmission, NAT, nucleic acid test, PCR, polymerase chain reaction, PMTCT, prevention of mother-to-child transmission, RNA, ribonucleic acid, SABMR, South African Bone Marrow Registry, SANBS, South African National Blood Service, TNC, total nucleated cell, UCB, umbilical cord blood, WMDA, World Marrow Donor Association

## Abstract

The probability of a Black African finding a matched unrelated donor for a hematopoietic stem cell transplant is minimal due to the high degree of genetic diversity amongst individuals of African origin. This problem could be resolved in part by the establishment of a public cord blood (CB) stem cell bank. The high prevalence of human immunodeficiency virus (HIV) amongst women attending antenatal clinics in sub-Saharan Africa together with the risk of mother-to-child transmission increases the risk of transplant transmissible infection. In addition to screening the mother in a period inclusive of 7 days prior to the following delivery, we propose that all CB units considered for storage undergo rigorous and reliable screening for HIV. The Ultrio-plus^®^ assay is a highly specific and sensitive test for detecting HIV, hepatitis-B and hepatitis-C viruses in peripheral blood. We validated the Ultrio-plus^®^ assay for analytical sensitivity in detecting HIV in CB at the level of detection of the assay. Until more comprehensive and sensitive methods are developed, the sensitivity and reliability of the Ultrio-plus^®^ assay suggest that it could be used for the routine screening of CB units in conjunction with currently recommended maternal screening to reduce the risk of transplant transmissible infection.

## Introduction

Allogeneic haematopoietic stem cell transplantation (HSCT), using cells harvested from bone marrow, peripheral blood or umbilical cord blood (UCB), is used for the treatment of many haematological malignancies, non-malignant blood disorders and metabolic disorders. With regard to UCB, more than 7 30 000 units have been stored worldwide in public cord blood (CB) banks providing an immediate source of HSCs for allogeneic transplants with no associated risk to the donor [1].

One of the biggest challenges in the establishment of a public UCB bank in high human immunodeficiency virus (HIV) prevalence regions is to screen effectively for HIV prior to storage of a unit. Current international screening methods involve screening of the donor (mother) for infectious diseases and for other potential risk factors which would result in the rejection of a donated UCB unit if found to be positive. To date, no tests that detect HIV ribonucleic acid (RNA) have been validated for screening UCB units for infectious diseases.

### Prevalence of HIV infection in South Africa

Most countries have an estimated HIV prevalence below 5%; however, sub-Saharan Africa carries the greatest HIV burden with many countries having a prevalence of over 10%, with Botswana (22.2%), Lesotho (22.7%) and Swaziland (28.8%) being among the highest [2]. With a prevalence of 12.7%, South Africa is considered to have one of the greatest epidemics of HIV worldwide and is faced with enormous challenges in the areas of HIV prevention (including education) and treatment. With regard to the prevalence of HIV/AIDS in South Africa, the data from Statistics South Africa [3], reveal the following:

- The estimated overall HIV prevalence rate is 12.7%. The total number of people living with HIV is estimated at approximately 7 million. For adults aged 15–49 years, an estimated 18.9% of the population is HIV-positive.
- During 2015, approximately 6.7 million people aged 15 and older and approximately 2 40 000 children were infected with HIV.

**Fig. 1.**
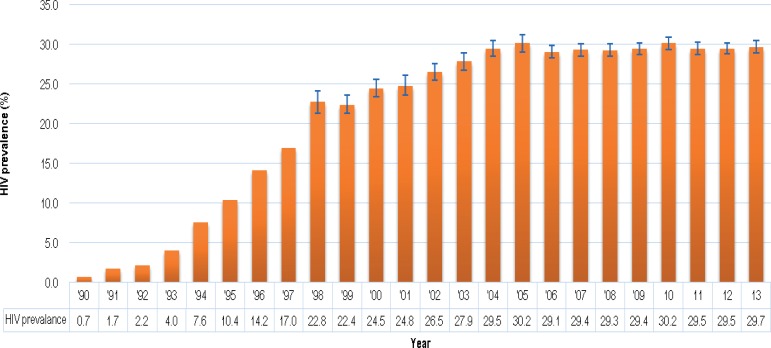
HIV prevalence trend among antenatal women, South Africa, 1990–2013. *Source*: [4], National Department of Health, 2015.

Of particular importance for establishing a public UCB bank is the high rate of HIV infection among pregnant women, from whose placentas UCB units would be obtained after birth. According to the 2013 National Antenatal Sentinel HIV Prevalence Survey in South Africa, the national HIV prevalence for a woman attending antenatal clinics in 2013 was estimated at 29.7% (95% CI 28.8–30.2). The HIV prevalence trend from 1990 to 2013 among women attending antenatal clinics is indicated in Fig. 1.

It appears that the sharp increase in the antenatal HIV prevalence from the early 1990s has levelled out since 2004 and has remained more or less stable at around 29% in recent years. These high prevalence rates would result in the disqualification of significant numbers of potential UCB units even before collection. This underscores the importance of pre-screening questionnaires for the mothers so that only potentially usable UCB units are collected and unnecessary downstream screening expenditure is prevented.

Mycobacterium tuberculosis (TB) is not routinely tested for on blood donation. There is no published report of transfusion-transmission of TB even though the organism is blood-borne. The general recommendation is that individuals with tuberculosis defer for 2 years following confirmation of cure [5].

### Probability of obtaining HIV-positive umbilical cords: vertical transmission of HIV from mother to child

HIV infection and transmission can occur *in utero* and is termed mother-to-child transmission (MTCT), vertical transmission or trans-placental transmission [6]. In developed countries, the prevalence of HIV MTCT in infected women who have not received antiretroviral treatment ranges between 13% and 32%, while it increases to between 25% and 48% in developing countries, with 30% of these HIV-positive infants being infected *in utero* [7]. A study done by Taha *et al.* [8] furthermore suggested that the rate of *in utero* infection increases in newly infected mothers at a frequency of 17.8%, as opposed to mothers infected earlier who had an *in utero* transmission of 6.7% (*p* < 0.0001) [8]. In addition, *in utero* transmission is positively associated with hard drug use during pregnancy, maternal antenatal viral load (VL), lack of maternal antiretroviral treatment during pregnancy and low birth weight [9]. Magder *et al.* also found that after controlling for VL and antiretroviral treatment, low birth weight remained significantly associated with *in utero* transmission. Following the prevention of mother-to-child transmission (PMTCT) of HIV programme in South Africa, in which HIV-positive pregnant women received triple anti-retroviral therapy (ART), the 2012 national PMTCT survey found a 2.7% national MTCT rate in pregnancy and intrapartum with a greater than fourfold differential range of rates across the nine provinces (1.4–5.9%). The failure to achieve complete elimination of MTCT can be ascribed, amongst other factors, to delayed antiretroviral initiation due to false-negative initial screening, primary HIV infection during pregnancy and maternal treatment failure due to inadequate adherence or drug resistance.

Studies that distinguish between true *in utero* infection, intrapartum infection (occurring during the time of birth) and perinatal infection (period around birth – between 5 months before and 1 month after birth) found that around 5–8% of HIV MTCT occurs *in utero*, while 15–30% occurs intrapartum when maternal antiretroviral treatment is not used [10]. With the use of antiretroviral therapy, the rate of both *in utero* and intrapartum transmission has been significantly reduced; however, the relative rate of *in utero* infection appears to have increased over time [9, 11]. Possible explanations include a reduction in intrapartum transmission due to enhanced PMTCT prophylaxis, resulting in a proportionate increase of *in utero*-infected infants and/or improved sensitivity of newer viral detection assays [9, 11].

Guevara *et al.* [7] stated that HIV RNA measurements from maternal and CB plasma allow for the quantitative assessment of HIV viraemia in the mother and infant, respectively. This statement was further supported by Biggar *et al.* [12] who conducted polymerase chain reactions (PCR) on infants to detect the HIV genome. In these studies, the infants were only considered to be infected with HIV *in utero* if HIV was detected by PCR on UCB. Townsend *et al.* [13] found in their study on mothers receiving ART that three infants (from a total of 2117 infants born) contracted HIV from their mothers despite the mothers being on ART and having VLs below 50 IU/mL. Two of these infants showed evidence of *in utero* transmission.

### International regulatory standards for screening of UCB units

When a UCB unit is received at a processing facility, it undergoes rigorous screening in order to meet the requirements for transplantation. Detailed information about the unit – e.g. total nucleated cell count, human leukocyte antigen (HLA) typing and specific tests performed on the UCB unit and/or mother are all recorded in a ‘Cord Blood Unit Report’ [14].

CB banks apply maternal health questionnaires that serve as a pre-screening tool which aims, *inter alia*, to identify certain risk factors related to determining the suitability of the unit for transplantation, prior to acceptance or storage. Requirements to identify these risk factors vary among different CB banks. The list may cover various blood disorders (red and white blood cells and platelets), certain genetic disorders (including monogenic disorders), cancers (Leukaemia's), metabolic disorders, severe autoimmune disorders and infectious diseases.

### Screening UCB units for infectious diseases

There are currently three potential ways to screen for infectious diseases: (1) screening the mother 7 days prior to or after delivery; (2) re-testing maternal donors at a 6-month follow-up; and (3) testing the UCB unit.

#### Maternal screening

The minimal evaluation for infectious agents is performed through serologic screening and nucleic acid testing (NAT) of the maternal sample as a proxy for the UCB unit [15].

**Fig. 2.**
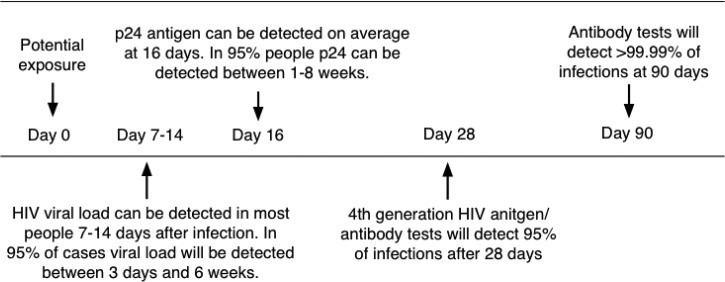
Average time after exposure to detect HIV antigens and antibodies. *Source*: [16] HIV-i-Base, 2013.

Pregnant mothers are tested for HIV by serological methods at the time of their first antenatal visit. Due to these tests having varying window periods of detection, they have limited value in ruling out the possibility of the patient being HIV positive. The 4^th^ Generation ELISA (enzyme-linked immunosorbent assay) is currently the most reliable of the serological tests with a window period of approximately 16 days–4 weeks (Fig. 2; [16]). This test would be preferable to a rapid test such as the ABON™ HIV 1/2/O Tri-Line Human Immunodeficiency Virus Rapid Test which is currently used in healthcare facilities and has a much greater window period of up to 6 weeks. Therefore, if a patient tests HIV-negative at the first screening during pregnancy, this does not rule out the possibility that she may have been in the window period of infection at the time of her last screening or that she may still contract HIV during her pregnancy. At present, mothers that consent to UCB donation are screened again for infectious diseases – including HIV – within 7 days prior to or after delivery. Some CB banks also require an additional follow-up screening of mothers 6 months after delivery. In such a case, a UCB sample would only be eligible for further consideration if the screening results for all the time periods were negative, necessitating the placement of the UCB unit in quarantine for this period of time. Recently released national consolidated guidelines for the PMTCT of HIV and the management of HIV in children, adolescents and adults suggests that in the case of HIV-negative mothers, HIV testing should be repeated 3 monthly during pregnancy, at labour/birth, at the 6-week postnatal/EPI (expanded programme of immunisation) baby visit and 3 monthly whilst breastfeeding [17].

Although there are benefits to conducting follow-up screening on the mother, it places an administrative burden on the CB bank. In addition, it is often difficult to locate patients and many might not stay close to the hospital or clinic. The onus of re-testing the mother lies on the bank and the bank would, therefore, be responsible for any additional costs involved for the patients to return to the hospital or clinic for screening.

#### UCB unit screening

An alternative, and possibly more reliable strategy, would be to screen the mother at the time of delivery as well as the UCB unit once collected. Although screening of the UCB unit is recommended by NetCord-FACT [18] many test kits (for infectious diseases) have not been approved by the Food and Drug Administration (FDA) for use on UCB. In the case where a screening test – which is not accredited for UCB – is used, the UCB bank is advised to denote the outcome and annotate that the test has not yet been validated [18].

The NetCord-Foundation furthermore requires that each CB unit should be tested for evidence of infection for the communicable diseases listed in Table 1, using licensed donor screening tests when available, prior to the release of the UCB unit.

**Table 1.** Communicable diseases which require testing prior to the release of a UCB unit, according to the NetCord Foundation Accreditation Manual [18]

Communicable diseases which require testing for prior to the release of a UCB unit
Human immunodeficiency virus type 1 Human immunodeficiency virus type 2 Hepatitis B virus Hepatitis C virus Human T cell lymphotropic virus type 1 Human T cell lymphotropic virus type 2 Treponema pallidum (syphilis) Cytomegalovirus Any additional agents required by Applicable Law at the time of the release of the UCB unit

The reluctance to standardise screening of UCB units stems from concerns about reducing the volume of the UCB unit as a result of additional testing requirements. Measurements might also be affected by dilutions with anti-coagulant in the collection bags. Furthermore, if appropriate provision for testing and re-testing is not made, it might require thawing of the UCB unit, which could damage the integrity of the sample [18]. However, in order to overcome this last-mentioned logistical issue, small segments attached to the UCB bag can be sealed off and frozen together with the UCB unit during sample processing. These segments are representative of the UCB unit and can easily be separated and used for additional screening or sample analyses without compromising the UCB unit's integrity or volume.

It seems therefore that the most feasible option, which would also be the most stringent in screening for infectious diseases, would be to screen the UCB unit in addition to the maternal sample, the latter being screened seven days prior to or after delivery.

#### Stringency in screening and acceptance criteria

The heavy burden of HIV disease in South Africa combined with the risk of MTCT highlights the risk of transplant transmissible infections. UCB banks make their UCB units available to patients globally. These risks might, therefore, discourage potential international registries from using UCB units that originate from southern African UCB banks. This would have important financial consequences and might render any such bank non-viable.

In order to increase the stringency of detection methods for infectious diseases, tests that have been validated for use on peripheral blood also need to be validated/verified for use on UCB. This would increase screening comprehensiveness and improve confidence in the quality of the UCB units. By only screening the donor (mother) for infectious diseases, certain HIV-infected UCB units could go undetected. Performing screening on both the maternal sample and UCB unit would, therefore, increase the safety of the product.

Another point to consider would be if the VL of a mother receiving ART is below 50 IU/mL and the subsequent screening of the collected UCB unit is negative, whether this UCB unit should be discarded, made available to the general public (seeing that the test result is negative) or be stored separately for potential use in HIV-positive patients? This could be done with risk assessment scores of the mother together with stringent and sensitive testing using NAT/PCR on the UCB units to ensure the reliability of results. It is, however, noteworthy that full suppression of VL has been observed when employing molecular methods to detect HIV RNA in patients on ART [19, 20]. Many HIV-negative individuals would probably not be comfortable with receiving a UCB unit (albeit negative) from an infected mother regardless of her current health status. In these cases, it might be necessary to keep these samples separate from samples that are negative for both maternal and UCB unit screening for potential use in HIV-positive patients. Indeed, as the gap between patients requiring kidneys for transplantation and the potential donor pool widens, the idea of using kidneys from HIV-positive donors for HIV-positive patients is a valid option [21].

**Table 2.** Ultrio-Plus^®^ assay screening results for 10 HIV spiked UCB units

Number of reactive tests per dilution				
Unit no.	01:02	01:04	01:08	Total
	46 IU/mL	23 IU/mL	11.5 U/mL	
1	10	5^a^	10	25
2	10	10	10	30
3	10	10	10	30
4	10	10	10	30
5	10	10	10	30
6	10	10	10	30
7	10	9^b^	10	29
8	10	10	10	30
9	10	10	10	30
10	10	10	10	30

a Invalid reactions due to inadequate sample volume.

b Invalid analysis due to sample error code related to instrument mechanics.

In a country as severely affected by HIV as South Africa, consideration could be given to creating a separate storage facility that would store UCB units collected from mothers who have a history of infectious diseases and/or used ART but in whom VL is undetectable and both maternal sample and UCB unit tested PCR negative. This would potentially allow the utilisation of about 25% more of the UCB units available for collection.

A critical component of clarifying these concerns will be the accuracy and sensitivity of tests used to detect the various infectious diseases.

### Ultrio-plus^®^ assay

The Ultrio-Plus^®^ assay is used internationally by blood centres [including the South African National Blood Service (SANBS)] for the screening of HIV, hepatitis B and hepatitis C viruses (HBV and HCV), and has recently been validated for specificity and sensitivity in peripheral blood samples [22]. It is a qualitative *in vitro* nucleic acid test (NAT) used for the simultaneous detection of HIV-1 ribonucleic acid (RNA), HBV deoxyribonucleic acid (DNA) and HCV RNA in human peripheral blood, bone marrow and cadaveric tissue (using plasma or serum). The test is developed, manufactured and distributed by Hologic, previously known as Gen-probe, in collaboration with Grifols (Grifol, Emeryville, CA) previously known as Novartis Vaccines and Diagnostics, Inc. It utilises target amplification nucleic acid probe technology and has an internal control incorporated for monitoring assay performance in each individual specimen. Although it does not discriminate initially between a positive signal for HIV-1, HBV or HCV, the technique is fast, effective and accurate in determining which samples are contaminated with these infectious diseases and should, therefore, be discarded. Specimens found to be reactive in the Ultrio-Plus^®^ assay can be run in individual HIV-1, HCV and/or HBV discriminatory assays to determine if they are reactive for HIV-1, HCV, HBV or any combination of the three, should the need arise.

Should public UCB banks be established in high HIV prevalence regions such as South Africa, all UCB units would need to undergo compulsory routine infectious diseases screening to comply with international regulatory standards. It would be imperative to have a sensitive and reliable assay for the detection of HIV RNA in potential UCB units prior to banking. It would also be beneficial to use the same screening test for both maternal samples and UCB units for result comparison.

The Ultrio-Plus^®^ assay has been approved by the FDA for use on living donor blood samples as well as cadaveric blood specimens. However, there have been no reports in which the Ultrio-Plus^®^ assay has been validated for the screening of CB plasma. Such a validation would support the use of this assay in the routine screening of UCB units intended for UCB banking and subsequent transplantation.

#### Validation of the Ultrio-Plus^®^ assay for its sensitivity in detecting HIV-1 in UCB units

A pilot study, with the necessary ethics approval and informed consent, was conducted at the Steve Biko Academic Hospital (Pretoria, South Africa). In order to obtain a panel of UCB units for validation purposes, UCB plasma units were first screened using the Ultrio-Plus^®^ assay prior to spiking them with HIV-1, in order to confirm their HIV negative status. Ten UCB plasma units, which satisfied the volume requirements for the sensitivity analyses of the Ultrio-Plus assay, were subsequently spiked with three dilutions (1:2, 1:4 and 1:8) of an HIV-1 subtype C-positive quality control stock (diluted 1:80) with a known HIV VL (routinely used by the SANBS). The VLs of the dilutions were thus 46 IU/mL (1:2 dilution); 23 IU/mL (1:4 dilution); and 11.5 IU/mL (1:8 dilution). The Ultrio-Plus^®^ has a 95% limit of detection of 28.6 IU/mL. [The 95% and 50% level of detection is estimated by the manufacturer to be 27.5 IU/mL (21.7–39.5) and 6.3 IU/mL (5.0–7.4)] [22]. In the present study, the HIV VL dilutions were decided upon in order to investigate the sensitivity of the assay below the reported number of copies (for detection of HIV-1 at 11.5 IU/mL).

#### Results

Nine of the UCB units delivered adequate volumes of UCB plasma to perform 30 repeat tests. One UCB unit yielded sufficient UCB plasma to perform 25 of the 30 repeat tests and is included in the results presented in Table 2. For the total of 295 samples, 289 were reactive. Five samples from patient 1 (for the 1:4 dilution) could not be run due to inadequate sample volume while an instrument error was detected for one sample from patient 7. If these six samples are not taken into account, the test had 100% detection rate of HIV-1 at a VL of 11.5 IU/mL.

### Conclusion

The South African Bone Marrow Registry (SABMR) is one of two such registries in Africa, with up to 70% of the registered donors being Caucasian [23]. The high degree of genetic diversity and the low number of Black Africans who are registered with the SABMR collectively decrease the probability of finding a matched unrelated donor for these individuals. It is believed that establishing a public CB bank would help alleviate the shortage of compatible units by being representative of South African demographics, and could also service the unmet needs of the genetically diverse African population worldwide for the treatment of haematological and non-haematological diseases. The feasibility and public acceptance of establishing a public CB bank in South Africa have been investigated with results indicating favourable support from the community [24, 25]. In addition, a strategy has been proposed for the basis on which a public CB bank would need to be constituted to accommodate the diversity of local and regional populations [26].

The ethical issues related to CB banking involve mainly private banking. Public banks receive CB units altruistically from donors and these are listed on international registries. They are available for any potential recipient pending an adequate HLA match. Private banks on the other hand store CB for the exclusive future use by the donor or a matched relative. The limited use of private CB units as seen with low recall rates together with the profit-driven motive of private banks raises ethical concerns.

It is necessary that all UCB units intended for storage in a UCB bank undergo infectious disease screening to comply with international regulatory requirements. The high prevalence of HIV amongst women in ante-natal clinics in South Africa as well as the risk of MTCT underscores the risk of transplant transmissible infections. Consequently, all UCB units considered for banking will need to undergo rigorous and reliable screening prior to acceptance for storage in order to increase the safety of the product.

The Ultrio-Plus^®^ assay is used internationally by blood centres (including the SANBS) and is a highly specific and sensitive test used for detecting HIV-1 RNA, HBV DNA and HCV RNA in peripheral blood. The manufacturer of the assay reports on the assay's sensitivity in detecting HIV-1 subtype B panels; however, the vast majority of HIV-1 infections in South Africa are subtype C. The levels of detection by the Ultrio-Plus^®^ using an HIV-1 subtype B and subtype C standardised dilution panel were recently determined by Vermeulen *et al.* [22]. The authors found that the results for the subtype C panel were in line with the values provided by the manufacturer. In the present study, the Ultrio-Plus^®^ assay was validated for its analytical sensitivity in detecting HIV-1 in UCB units. To our knowledge, this is the first such validation to be performed using this assay. Confirmatory tests carried out on UCB from HIV-positive mothers would ensure that HIV-positive specimens will react in the same manner as the HIV-spiked samples. To date, it has not been possible to undertake an assessment of this nature due to the large number of CB samples that would need to be screened in order to detect a single positive unit. In light of the fact that the 2012 National PMTCT survey found a 2.7% national MTCT rate, this means that approximately 400 CB samples from HIV-positive mothers on ART would need to be tested in order to obtain approximately 10 positive samples. This is beyond the scope of the present study but could form part of a comprehensive prospective study for high HIV prevalence regions which might be considering the development of a CB bank.

According to currently accepted standards and practices, the Ultrio-Plus^®^ assay has been shown in our study to be as sensitive in detecting spiked HIV-1 in UCB as it is for detecting HIV-1 in peripheral blood. Thus, the Ultrio-Plus^®^ assay was found to detect HIV in 100% of samples up to a lower detection limit of 11.5 IU/mL. Until more comprehensive and sensitive methods are developed to eliminate non-detection of HIV-1 in positive samples, the sensitivity and reliability of this assay strongly suggest that it could be used for the routine screening of UCB units intended for UCB banking and subsequent transplantation.

Our recommendation is therefore that screening of UCB units using the Ultrio-Plus^®^ assay should be done in conjunction with maternal screening. An important consideration would be that some mothers may not disclose their HIV status if on ART with an undetectable VL. These UCB units would then be issued to negative recipients as both mother and UCB could test negative. We would, therefore, recommend that antibody screening of the mother should be done in parallel with the Ultrio-Plus^®^ assay to prevent any UCB units being issued to negative recipients as a result of both mother and UCB unit testing negative. Finally, the notion of using HIV-negative UCB units from HIV-positive mothers on ART could be explored for HIV-positive patients requiring an allogeneic HSCT, for which a matching HIV-negative donor cannot be found. These units could be stored separately in a public CB bank.
